# Supplementary material for: Antigen Targeting of Porcine Skin DEC205+ Dendritic Cells
Source: Vaccines (Basel). 2022 Apr 26;10(5):684. doi: 10.3390/vaccines10050684 (PMC9147619; doi:10.3390/vaccines10050684)
Supplement: Supplementary file 1 [file vaccines-10-00684-s001.zip › Original blots and gels.pdf]

Figure 1b

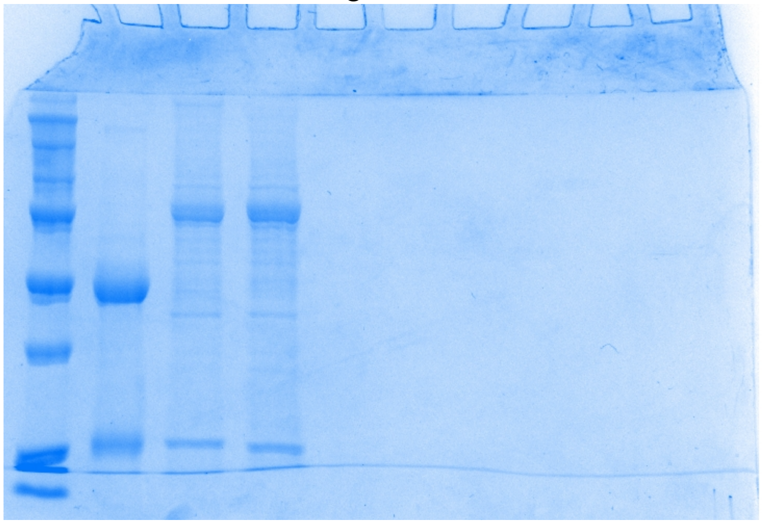

SDS-PAGE at 10% followed by Coomassie blue staining. Line 1: molecular weight ladder. Line 2: rAb ZH9F7. Line 3: rAb ZH9F7_Cap. Line 4: rAb 7DEU_Cap.

Figure 1c

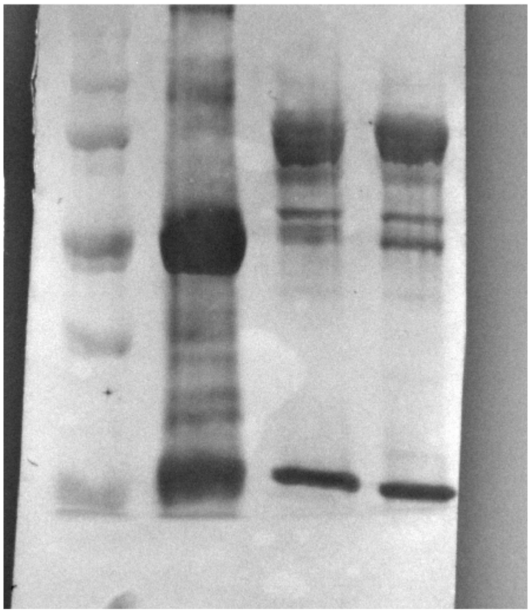

Western blot. Anti-porcine (H+L). Line 1: molecular weight ladder. Line 2: rAb ZH9F7. Line 3: rAb ZH9F7_Cap. Line 4: rAb 7DEU_Cap.
