# Supplementary material for: Antigen Targeting of Porcine Skin DEC205+ Dendritic Cells
Source: Vaccines (Basel). 2022 Apr 26;10(5):684. doi: 10.3390/vaccines10050684 (PMC9147619; doi:10.3390/vaccines10050684)
Supplement: Supplementary file 1 [file vaccines-10-00684-s001.zip › Supplementary legends.pdf]

Supplementary legends

Supplementary Table S1. Antibodies and dilutions used to flow cytometry staining.

Supplementary Figure S1. Identification of cDC1, cDC2 and monocytes DEC205^{+} from swine peripheral blood mononuclear cells. (a). Selection of the population of interest by size and complexity followed by doublet elimination. (b). Unstained cells. Once singlets were selected, the expression of CD14 was analyzed, identifying two different populations of CD14^{-} and CD14^{+} cells (monocytes). (c). Isotype controls. (d). Single CD14/FITC staining. (e). Double CD14/FITC + CD172/BV421 stained cells. CD14^{-} cells were analyzed for CD172a and CADM1 expression. (f). CD14/FITC + CD172/BV421 + CADM1/A647 panel allowed to identify cDC1 (CD14^{-} CADM1^{+} CD172a^{low}) and cDC2 (CD14^{-} CADM1^{+} CD172a^{high}) populations on PBMCs.

Supplementary Figure S2. FMO Identification of migratory cDC1, cDC2/LC and moDCs/MO from skin. (a). Skin APCs gated as FSC high and SSC high (P1), doublets eliminated (P2), and single cells (P3) selected for CD163, CD172a and CADM1 expression. (b). Cells from unstained control. (c). Isotype controls. (d). Single CD172a/FITC stained cells. (e). CD172a/FITC + CD163/BV421 stained cells. CD163^{high} CD172a^{+} cells were considered potential moDCs and macrophages (Macro). Then, CD163$^{-/low}$ CD172a$^{+/-}$ cells were selected and analyzed for CADM1 expression. (f). CD172a/FITC + CD163/BV421 + CADM1/A647 stained cells show the cDC1 (CD163^{-} CADM1^{+} CD172a^{-}) and cDC2/LC (CD163$^{-/low}$ CADM1^{+} CD172a^{high}).

Supplementary Figure S3. Expression of CD80/86 in different targeted populations. Histograms show the intensity of surface marker CD80/86 expression among different skin targeted populations.

Supplementary Figure S4. Identification of lymphocytes, DCs and Macrophages in superficial inguinal lymph nodes. (a). Strategy to analyze DEC205^{+}-targeted cells in superficial inguinal lymph nodes. After singlets were obtained (top panel), exclusion of B and T lymphocytes was carried out excluding the CD3^{+} CD21^{+} population. (b). Unstained control. (c). Isotype controls. (d). Single CD3/CD21/BV421 staining for lymphocytes exclusion. Then, the CD3^{-} CD21^{-} population was analyzed for CD163 and CADM1 expression. (e). CD3/CD21/BV421 + CD163/FITC stained cells. (f). CD3/CD21/BV421 + CD163/FITC+ CADM1/A647 stained cells allowed to identify the potential DCs (CD3^{-} CD21^{-}CADM1^{+} CD163^{-}) and macrophages (CD3^{-} CD21^{-}CADM1$^{+/-}$ CD163^{+}).

Supplementary Figure S5. Identification of CD4^{+}CD8^{-}, CD4^{-}CD8^{+} CD4^{+}CD8^{+} T lymphocytes stimulated *in vitro*. (a). After doublet elimination, the expression of CD4 and CD8 was analyzed for identification of the CD4^{+}CD8^{-}, CD4^{-}CD8^{+}, and CD4^{+}CD8^{+} populations. (b). Unstained cells. (c). Isotype controls. (d). Single CD4/A647 stain. (e). Single CD8a/FITC stain. (f). Double CD4/FITC + CD8a/A647 stain cells allowed to identify CD4^{+}CD8a^{-}, CD4^{-}CD8a^{+} and CD4^{+}CD8a^{+} populations. (g)-(i). FMO for dentification of IFN-γ positive cells on CD4^{+}CD8^{-} (g), CD4^{-}CD8^{+} (h), and CD4^{+}CD8^{+} (i).
