# Supplementary material for: Antigen Targeting of Porcine Skin DEC205+ Dendritic Cells
Source: Vaccines (Basel). 2022 Apr 26;10(5):684. doi: 10.3390/vaccines10050684 (PMC9147619; doi:10.3390/vaccines10050684)
Supplement: Supplementary file 1 [file vaccines-10-00684-s001.zip › Supplementary table S1.pdf]

Table S1. Antibodies and dilutions used to flow cytometry staining.

Antibody	Company	Dilution
Anti CD163 (mouse IgG1)	Bio-Rad, United Kingdom	1:100
Anti CD172a (mouse IgG1)	Bio-Rad, United Kingdom	1:100
Anti CADM1 (chicken IgY)	MBL International, USA	1:500
Anti CD14 (mouse IgG2b)	Bio-Rad, United Kingdom	1:200
Anti CD3 (mouse IgG1)	Southern Biotech, USA	1:100
Anti CD21 (mouse IgG1)	Southern Biotech, USA	1:200
Anti CD8α (mouse IgG2a)	Bio-Rad, United Kingdom	1:200
Anti CD4α (mouse IgG2b)	Bio-Rad, United Kingdom	1:100
Anti DEC205 (mouse x pig IgG1)	Homemade	1:100
Streptavidin PE-conjugated	Southern Biotech, USA	1:100
BV421-conjugated rat anti-mouse IgG1	(BioLegend, USA)	1:100
A647-conjugated goat anti-chicken IgY	Invitrogen, USA	1:500
FITC-conjugated goat anti-mouse IgG2b	SouthernBiotech, USA	1:100
A647-conjugated goat anti-mouse IgG2b	Invitrogen, USA	1:200
PE-conjugated mouse anti-pig IFN-γ	BD Pharmingen, USA	-
