# Supplementary figures and images for: Antigen Targeting of Porcine Skin DEC205+ Dendritic Cells
Source: Vaccines (Basel). 2022 Apr 26;10(5):684. doi: 10.3390/vaccines10050684 (PMC9147619; doi:10.3390/vaccines10050684)

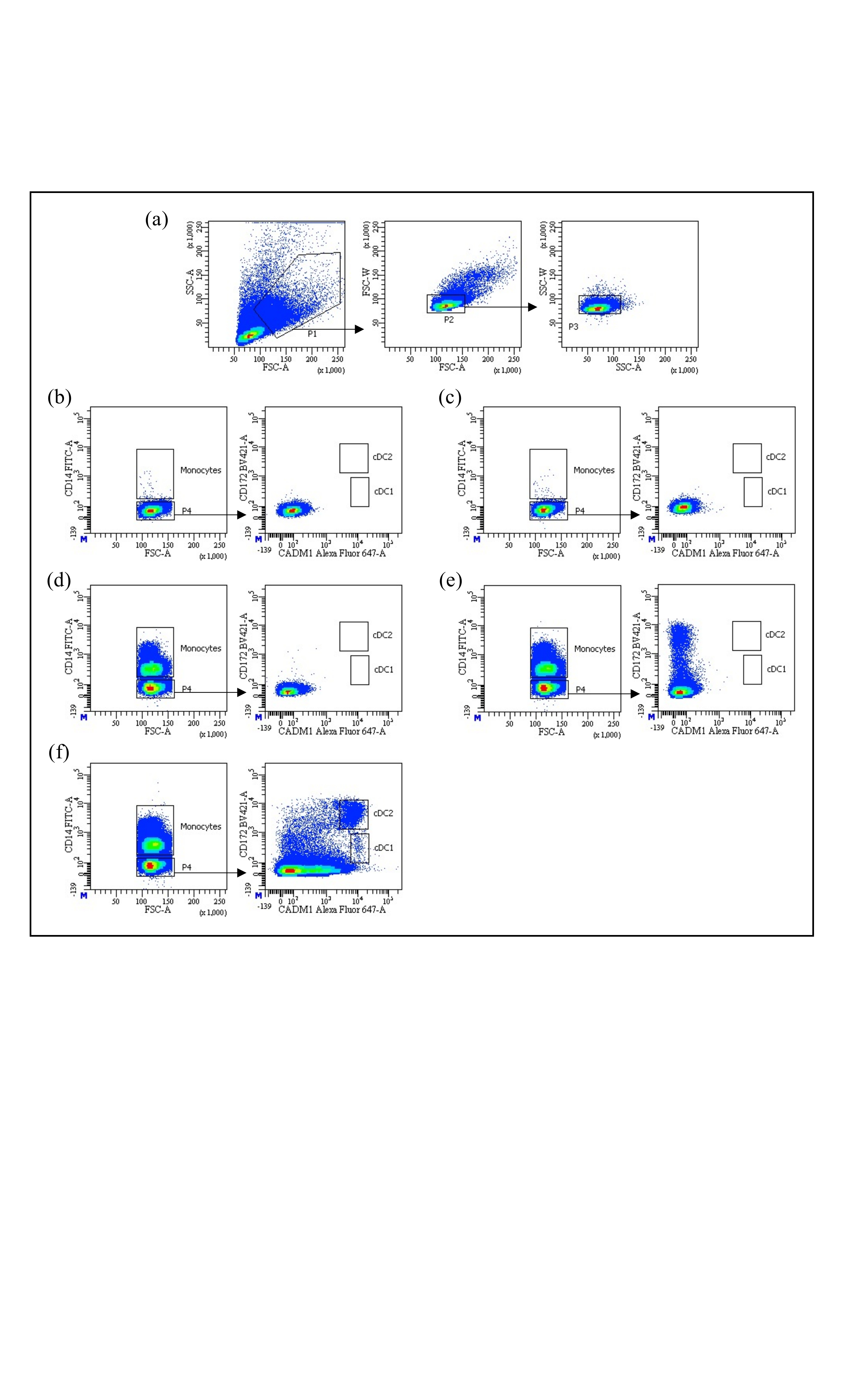

Supplement: Supplementary file 1 [file vaccines-10-00684-s001.zip › Supplemntary figure S1.jpg]

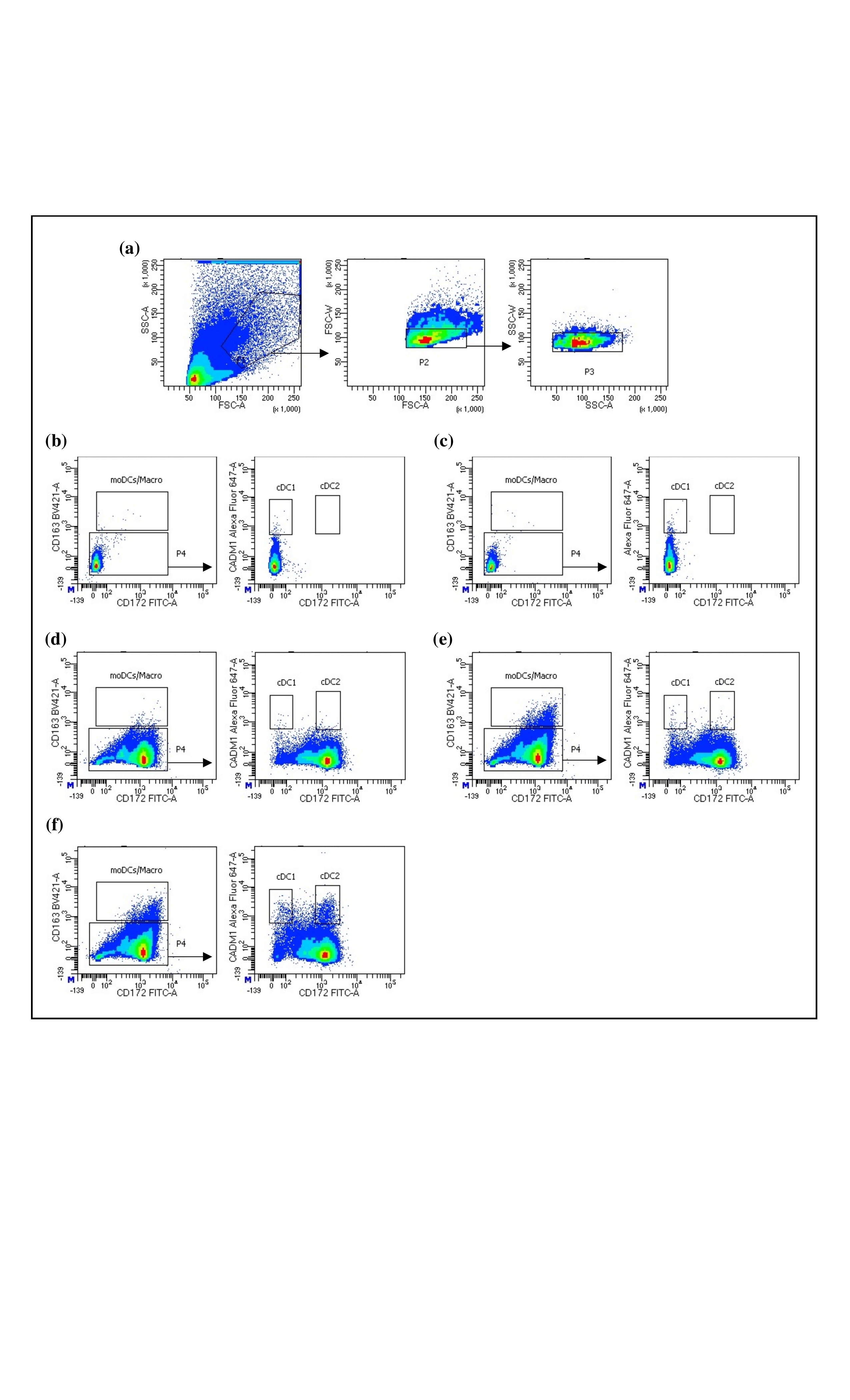

Supplement: Supplementary file 1 [file vaccines-10-00684-s001.zip › Supplemntary figure S2.jpg]

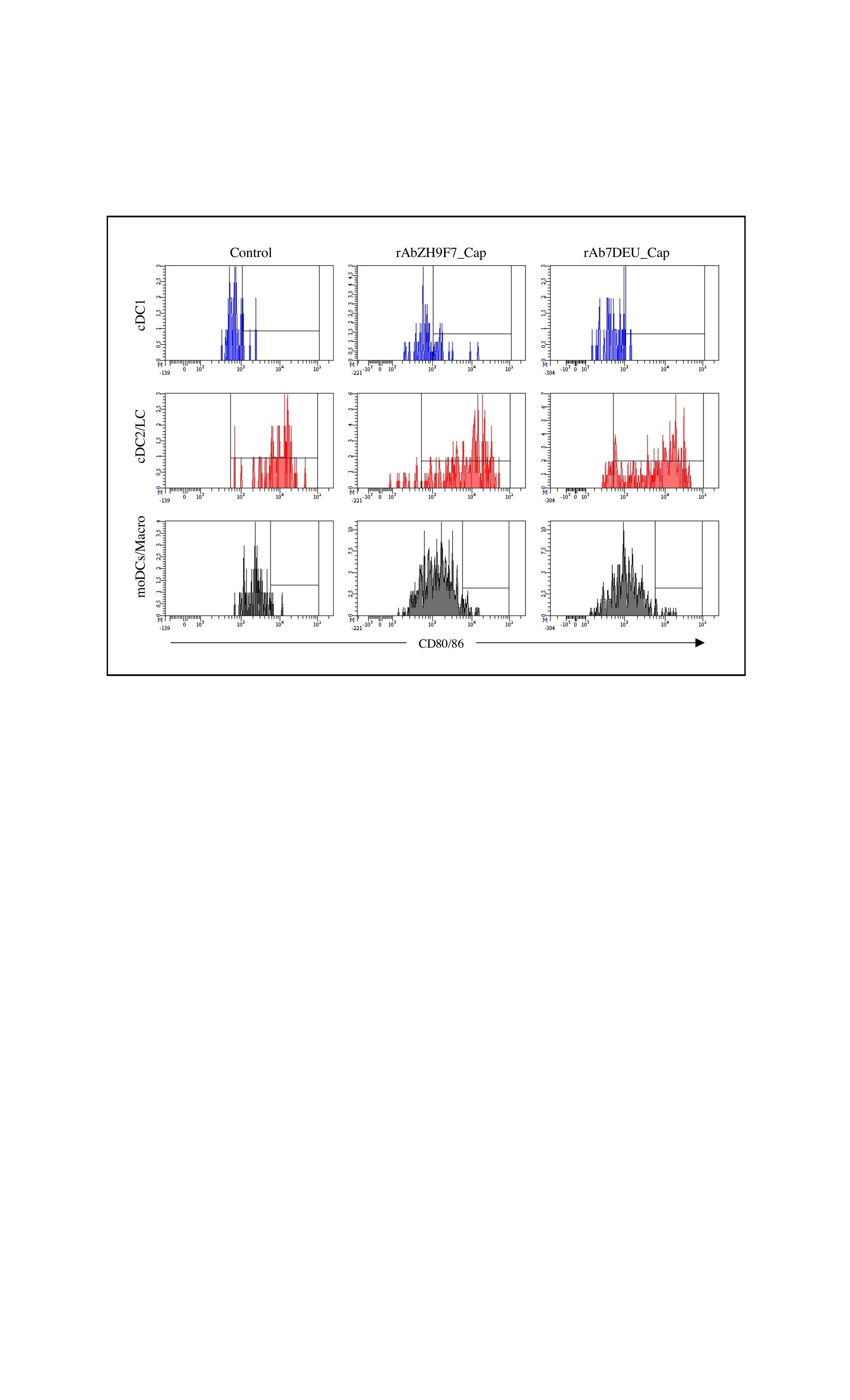

Supplement: Supplementary file 1 [file vaccines-10-00684-s001.zip › Supplemntary figure S3.jpg]

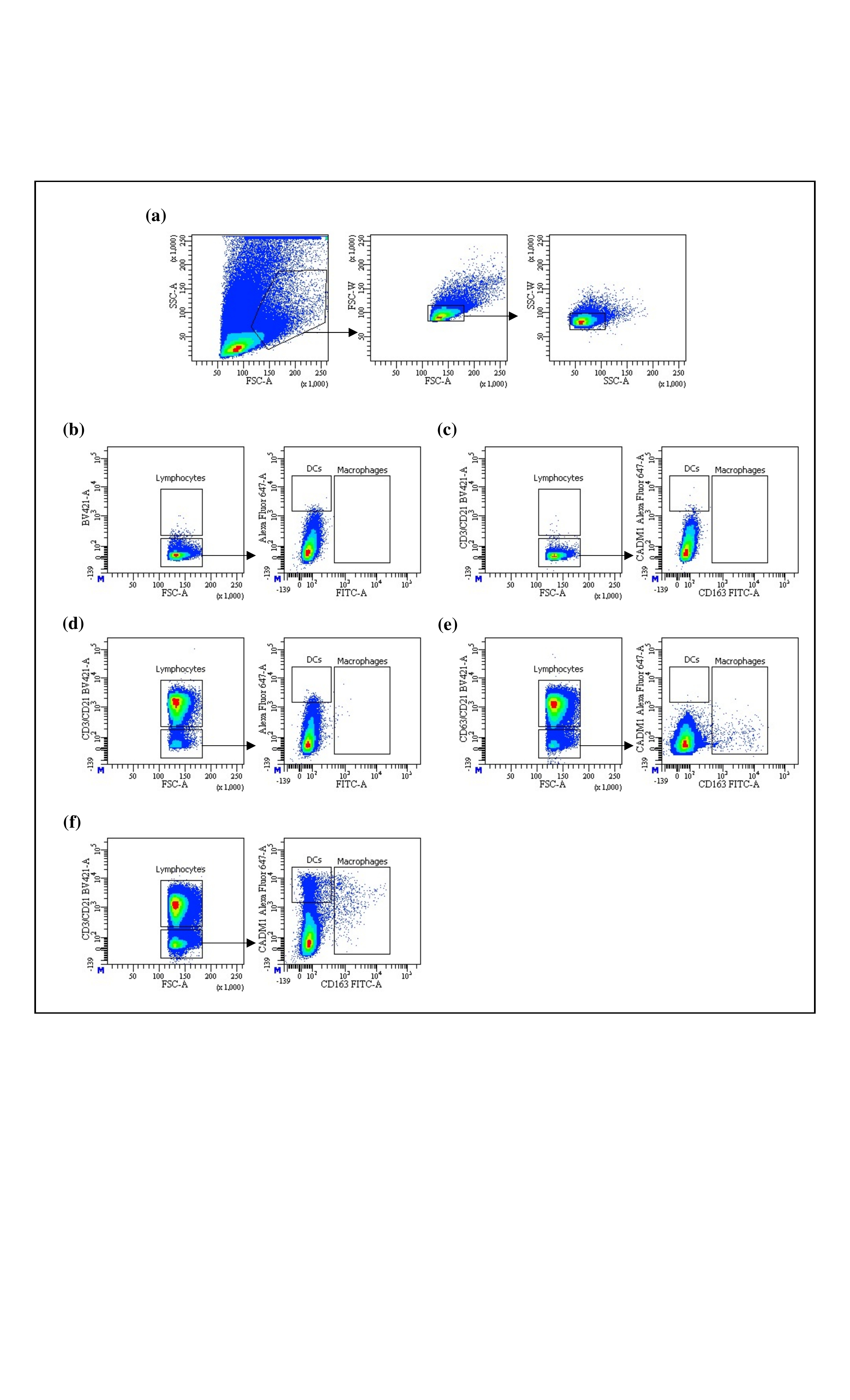

Supplement: Supplementary file 1 [file vaccines-10-00684-s001.zip › Supplemntary figure S4.jpg]

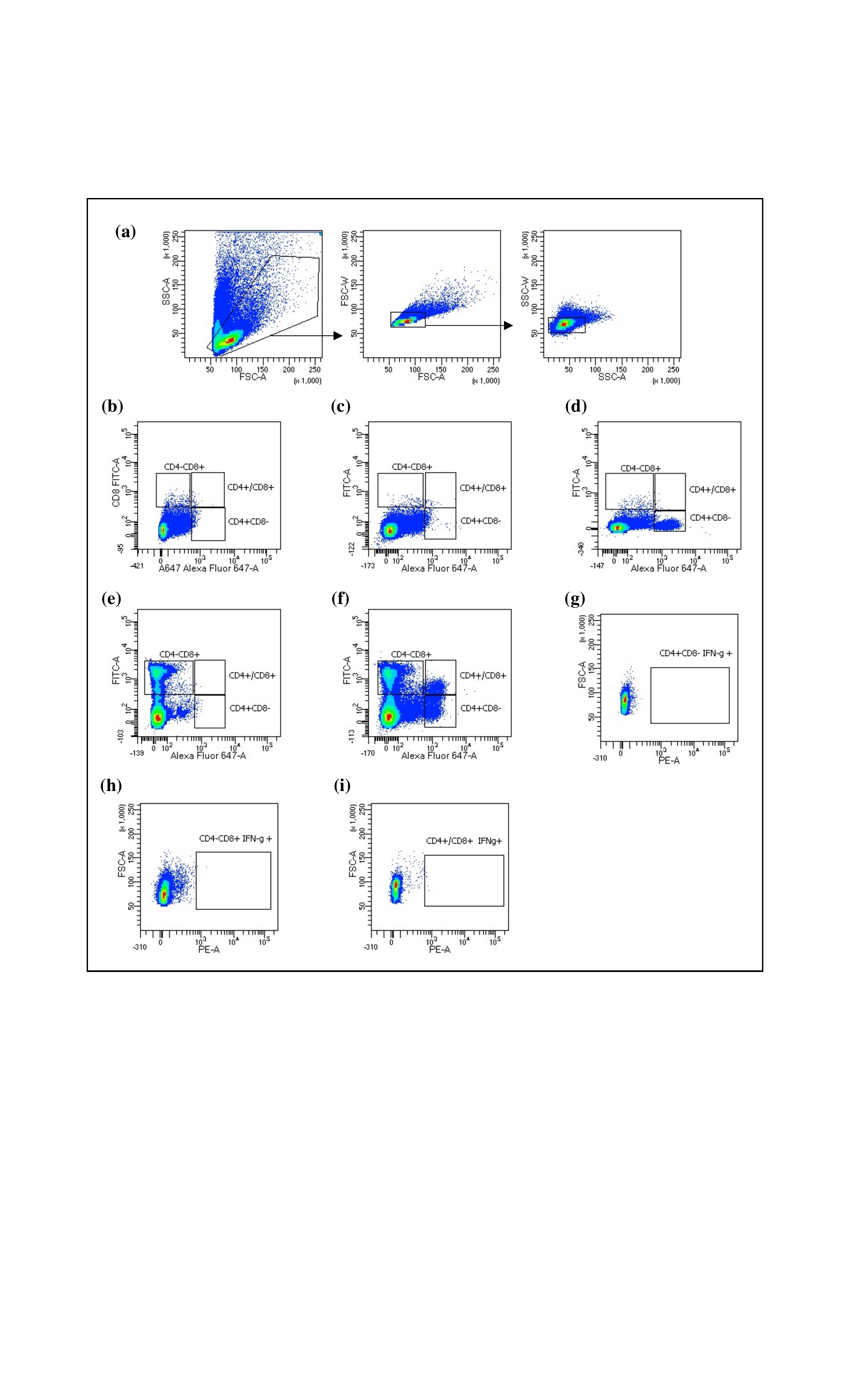

Supplement: Supplementary file 1 [file vaccines-10-00684-s001.zip › Supplemntary figure S5.jpg]
